# Lipoinjection and Multiple Internal Cuts for Congenital Constriction Bands: A New Treatment Approach

**DOI:** 10.1007/s00266-016-0744-4

**Published:** 2016-12-29

**Authors:** Yanko Castro-Govea, Amin Vela-Martinez, Luis Alberto Treviño-Garcia

**Affiliations:** Servicio de Cirugía Plástica, Estética, Maxilofacial y Reconstructiva, Hospital Universitario “Dr. José E. González”, Universidad Autónoma de Nuevo León, Av. Francisco I. Madero & Av. Gonzalitos s/n, Colonia Mitras Centro, 64460 Monterrey, Nuevo León México

**Keywords:** Congenital constriction band, Lipoinjection, Lipoinfiltration, “hourglass sign”, Congenital ring constrictions, Fibrous ring, Amniotic band

## Abstract

**Background:**

Traditional treatment for a congenital constriction band of the limb involves multiple Z-plasties and W-plasties. We propose an alternative surgical procedure for the treatment of congenital constriction bands that obviates the need for Z-plasties and eliminates the constriction band.

**Methods:**

We present the case of a 36-year-old woman with a congenital constriction band of the leg. Using a minimally invasive approach, the skin segment that included the band was dissected from the deep tissues. Afterwards, multiple slices were performed on the internal surface of the fibrous ring. This and lipoinjection were used to reverse the depression that characterizes the “hourglass sign” and homogenize the skin surface.

**Results:**

Results have remained stable in a follow-up period of 18 months.

**Conclusions:**

This surgical alternative can be considered as an option for the treatment of congenital constriction bands. It is a safe, reproducible procedure that does not cause additional scars and has good functional and aesthetic results.

**Level of evidence V:**

This journal requires that authors assign a level of evidence to each article. For a full description of these Evidence-Based Medicine ratings, please refer to the Table of Contents or the online Instructions to Authors www.springer.com/00266.

## Introduction

Congenital constriction bands (CCBs) are limb malformations that were described by Montgomery in 1832 [1]. Annular bands, amniotic bands, or Streeter dysplasia are also some terms used to describe this entity. Their incidence is variable, ranging from 1 in 15,000–1 in 50,000 [2]. Cases are usually sporadic, although a family history of this entity has been reported [3]. This defect usually presents with constriction rings in the distal part of the extremities or fingers (77%) [4], with a greater predilection for the arms [5]. In rare cases, there may be amputations, particularly when they are associated with craniofacial and orthopedic malformations.

The fibrous ring that causes tissue constriction usually has a groove of different depths and can be completely or partially circumferential [6]. Patterson [7] introduced a classification of CCBs, ranging from simple bands to intrauterine amputations. The origin of this congenital disorder remains unknown; however, there are two theories that attempt to explain it (intrinsic and extrinsic) [8, 9].

Regarding the treatment of CCBs, most reports in the literature use Z- and W-plasties as a standard, either once or twice [10–12]. In recent decades, there have been few contributions to the treatment of this disorder such as circumferential subcutaneous fat advancement flaps, rectangular plasties (Mutaf procedure), excision and direct closure, and triangular flap-plasty [13–16], perhaps in part because greater importance has been given to its functional aspect.

In this study, we describe an alternative surgical procedure for the treatment of congenital constriction bands, without removal of the fibrous band or the use of Z-plasty. Through a minimally invasive approach, we largely eliminate the contractile effect by making multiple cuts on the inside of the fibrous ring, and combine this maneuver with lipoinjection. A case report is presented with a follow-up period of 18 months.

### Surgical Technique

**Fig. 1:**
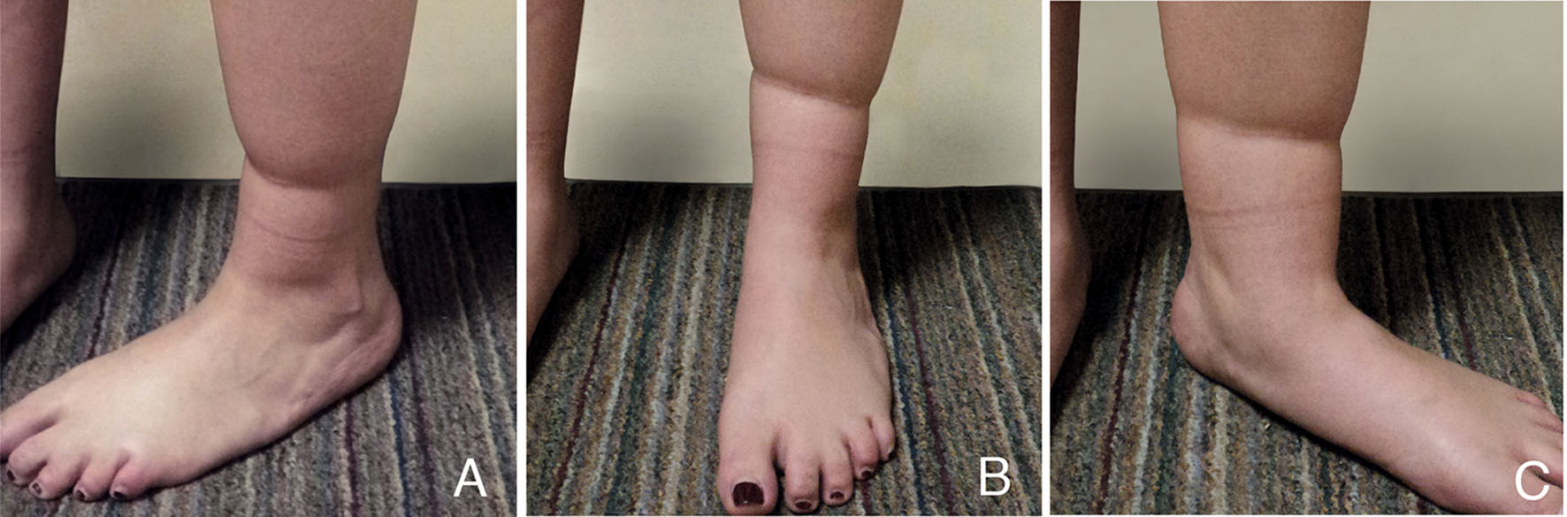
A 36-year-old woman with constriction bands. **a** Lateral view, **b**. frontal view, **c** medial view

The patient is a 36-year-old woman with no relevant medical history. She presents with a simple congenital constriction band in the distal part of both legs, without functional impairment (Type 1 Patterson Classification). The right leg had an incomplete circumferential constriction band with minimal depth and the left leg a circumferential constriction band of moderate depth (Fig. 1). The left leg was treated. The procedure was performed under epidural anesthesia with intravenous sedation.

### Removal of the Contractile Effect of the Band

**Fig. 2:**
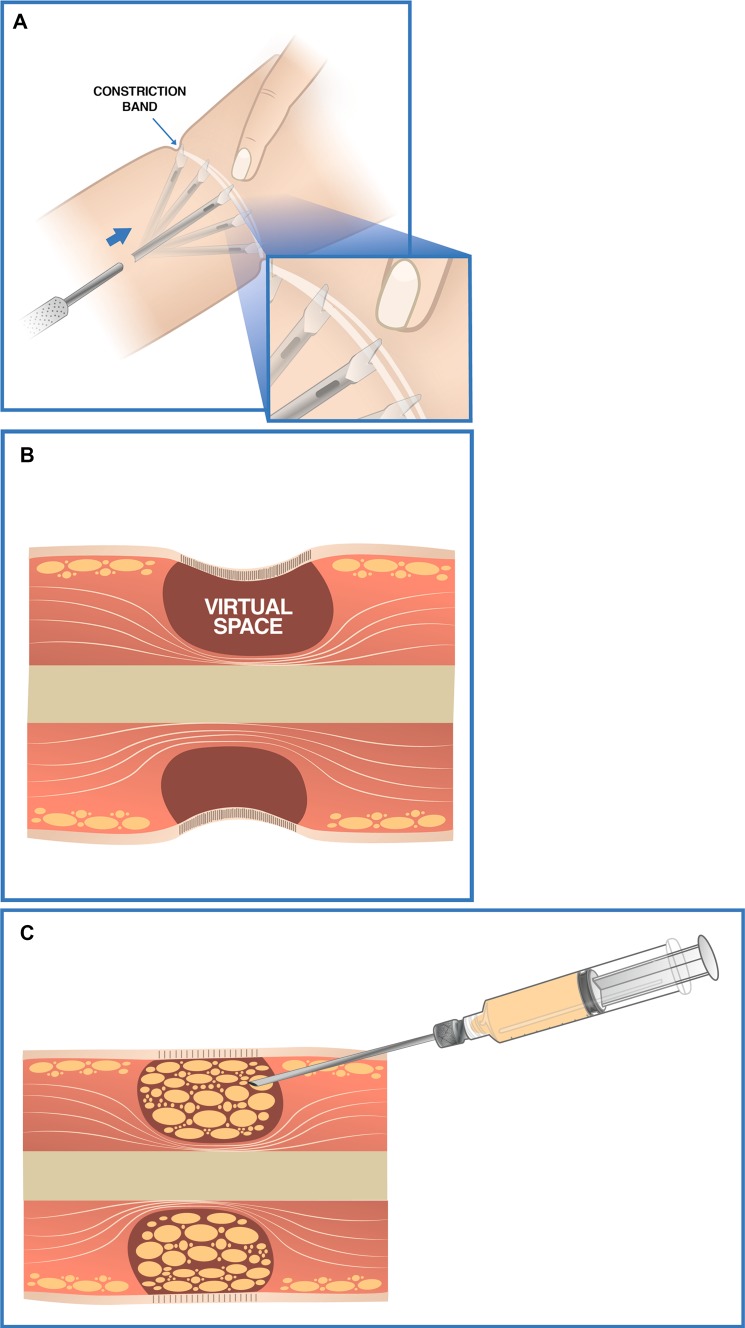
Surgical technique sequence. **a** Multiple perpendicular cuts performed in the inner surface of the constriction band. **b** Subcutaneous virtual space made by the detachment of the band from deep tissues and by the decrease of its contractile effect. **c** Tissue expansion and volumetric increase generated by lipoinjection in virtual space

Three approaches (2 mm) that were remote to the constriction band were marked. Initially, the fibrous band was released from the deep tissues with a flat-grove blunt-tipped 2-mm Toledo cannula, 10 cm in length. Afterwards, with this same cannula, multiple perpendicular cuts were made in the inner surface of the fibrous ring leaving a 1 cm gap between each cut until completing the circumference of the band. When cutting the band, it is important to not make external openings; a simple maneuver to avoid this is to place the index finger against one end of the tip of the cannula while controlling the depth of the cut (Fig. 2a). With this maneuver, the fibrous ring is weakened in its entirety; the ring diameter even increases due to elimination of the contractile effect caused by the multiple cuts. Dissection of the fibrous band from the deep tissue combined with attenuation of the contractile effect makes these tissues to discreetly lift like a tent, generating a virtual space that will be lipoinjected (Fig. 2b).

### Fat Tissue Collection and Lipoinjection

The periumbilical region is injected with 60 ml of a tumescent solution (250 ml Hartmann solution with 0.25 ml adrenaline), and 60 ml of fat is collected with a 3-mm blunt-tipped cannula attached to a 20-ml syringe. The fat obtained is prepared using the Coleman technique [17].

Lipoinjection is performed in the virtual subcutaneous space from a deep to a superficial plane with a 2-mm blunt cannula with 5-ml syringes (total 42 ml Fig. 2c). With lipoinjection, we generate volume and tissue expansion. This maneuver is not possible without separation of the band from deep tissue and without removal of the contractile effect. We inject the amount of fat needed to reverse the visual aspect of depression, without trying to overcorrect. The approaches should be about 5–7 cm away from the constriction band to prevent leakage of fat when it is injected. Finally, the approaches are sutured with 5–0 nylon, and a small segment of sterile strip is placed.

## Results

**Fig. 3:**
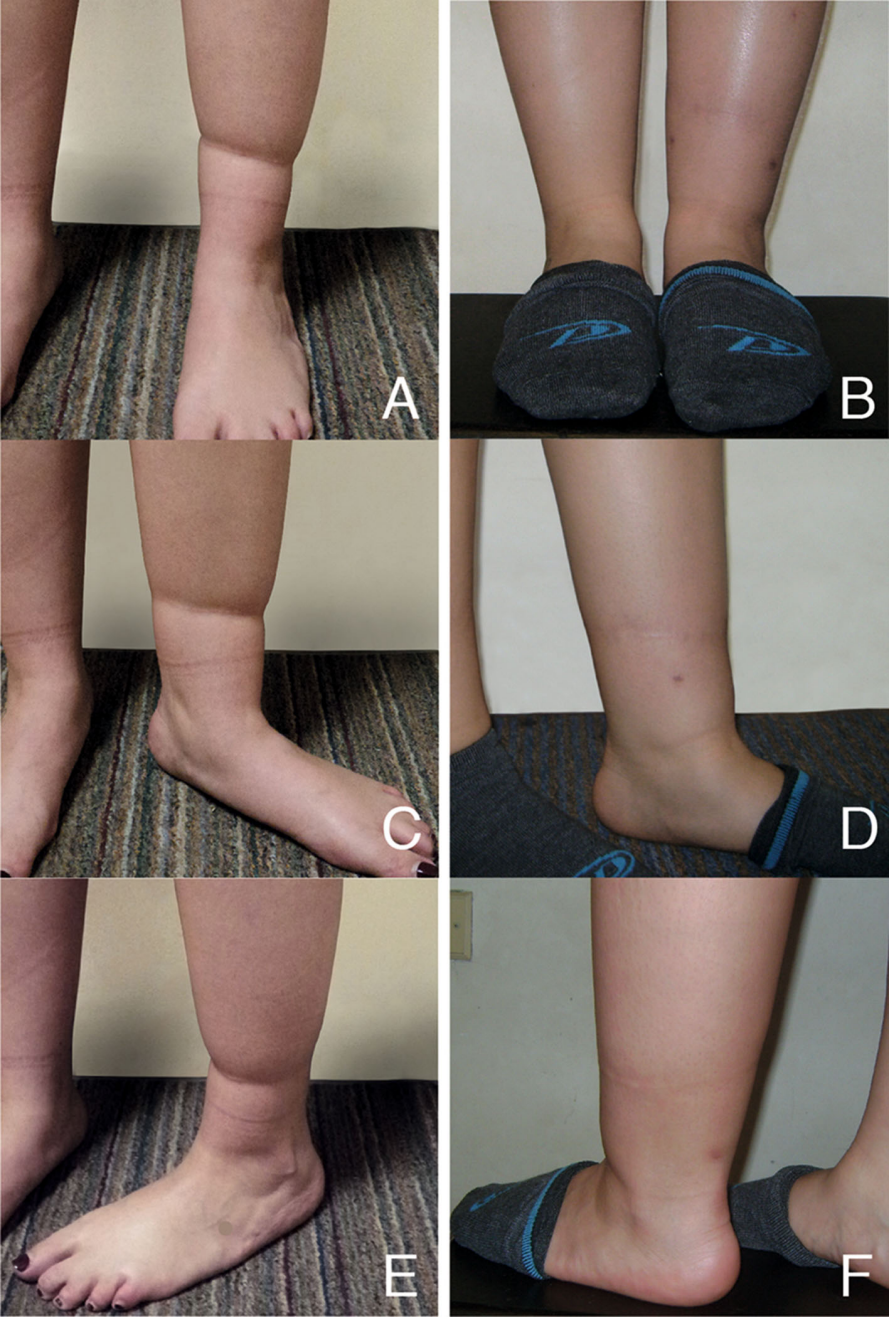
Change in the concave surface (“hourglass sign”) for a homogeneous surface. **a**, **c**, **e** preoperative view. **b**, **d**, **f** postoperative view 18 months after the procedure (notice the three approaches with some degree of hyperpigmentation)

The results of this technique have remained largely stable and were documented photographically in a follow-up period of 9–18 months (Fig. 3). There were no complications.

## Discussion

The functional and aesthetic appearance produced by CCBs is proportional to the degree of severity, and hence the relevance of personalizing each case. Regardless, we believe that treatment must not only provide good results functionally, but also a good aesthetic appearance because in fact, in most cases, this variable is usually the main reason for consultation.

After resection of CCBs, the skin closure techniques that are usually reported in the literature are Z-plasty and W-plasty [10–12]. Mutaf and Sunay designed deepithelialized and non-deepithelialized rectangular flaps elevating and opposing them in an alternating pattern on both sides of the groove; the groove is filled with turned-over dermo flaps [14].

Choulakian and Williams reported good results with excision of the CCBs and direct closure. In circumferential constriction bands, they recommend a two-step procedure[15] . Recently, other authors have modified standard techniques [16, 18–20]. The results reported with these techniques are satisfactory; however, they involve more scars. If we consider that the appearance of a constriction band is very similar to that of a scar, from our perspective, a surgical alternative that considers aesthetic appearance is also desirable.

Whatever the technique used for closure, it should strategically prevent recurrence of the depression characterized by the “hourglass sign” because soft tissue near the constriction band is atrophic; therefore, it would be prudent to have an approach that mostly increases rather than removes tissue.

In an attempt to solve this condition conservatively without losing functionality and pondering its aesthetic appearance, we propose this alternative surgical innovation. With multiple symmetrical cuts along the inner surface of the constriction band, we largely free the constrictive effect on soft tissues. In addition to this, the previous separation of the band from deep tissues produces a more favorable condition so that lipoinjection may have a greater effect on tissue compliance that allows changing a concave surface for a largely homogenous surface (Fig. 3). Even with attenuation of the contractile effect of the band, this tissue is not susceptible to overcorrection. With the volumetric increase generated by fat, the depression is reversed and its recurrence is prevented.

## Conclusion

We consider that the innovative aspect of this technique is to avoid resection of the CCBs as well as additional scars using truly limited approaches, and to even reduce, to some degree, the visual evidence of the fibrous band. This technical innovation is definitely a minimally invasive approach, which allows quick recovery and a return to everyday life and work. We believe that this option meets the criteria of good functionality and good aesthetics with a more natural appearance. It is a safe option that is easily reproducible and can be performed in an average time of 1.5 h, which makes it economical.

An important factor to consider is the variability of resorption of the injected fat. However, if necessary, fat injection can be repeated as an isolated procedure. Finally, it is important to comment that more studies are needed to reinforce the stability of long-term results.
